# Allergic Contact Dermatitis at the Incision Site After Surgical Casting

**DOI:** 10.7759/cureus.96598

**Published:** 2025-11-11

**Authors:** Hüseyin Kürüm, Omer Esmez

**Affiliations:** 1 Orthopaedics and Traumatology, Ergani State Hospital, Ministry of Health, Diyarbakır, TUR; 2 Orthopaedics, Elazığ Fethi Sekin City Hospital, Elazığ, TUR

**Keywords:** allergic contact dermatitis (acd), cotton casting, post trauma, synthetic cotton, ulna shaft fracture

## Abstract

Allergic contact dermatitis (ACD) may develop after postoperative casting. A 52-year-old male patient presented to our outpatient clinic complaining of pain, swelling, and bruising in his left forearm following a simple fall. An X-ray revealed a fracture of the left ulnar shaft. The patient, whose general condition and follow-up findings were good, was discharged on the first postoperative day with appropriate recommendations and instructed to return for a follow-up visit on the 15th day. Consequently, the possibility of ACD should be considered when using materials for casting, and it should be noted that even the casting cotton used can cause this condition.

## Introduction

Allergic contact dermatitis (ACD) may develop after postoperative casting. ACD is one of the most common occupational diseases in developed countries [1]. Certain groups are at a higher risk of developing ACD, and this risk appears to result from both genetic predisposition and environmental exposure [2, 3]. ACD localized to the plastered area may be caused by contact with components of the casting materials [4]. This case describes a male patient who developed ACD around the incision site in the left forearm region following cast application after undergoing plate osteosynthesis for an ulna shaft fracture. Although contact dermatitis following plaster application is rare, trauma to soft tissue, the cast material used, or both may have contributed to the development of contact dermatitis in this case.

## Case presentation

**Figure 1:**
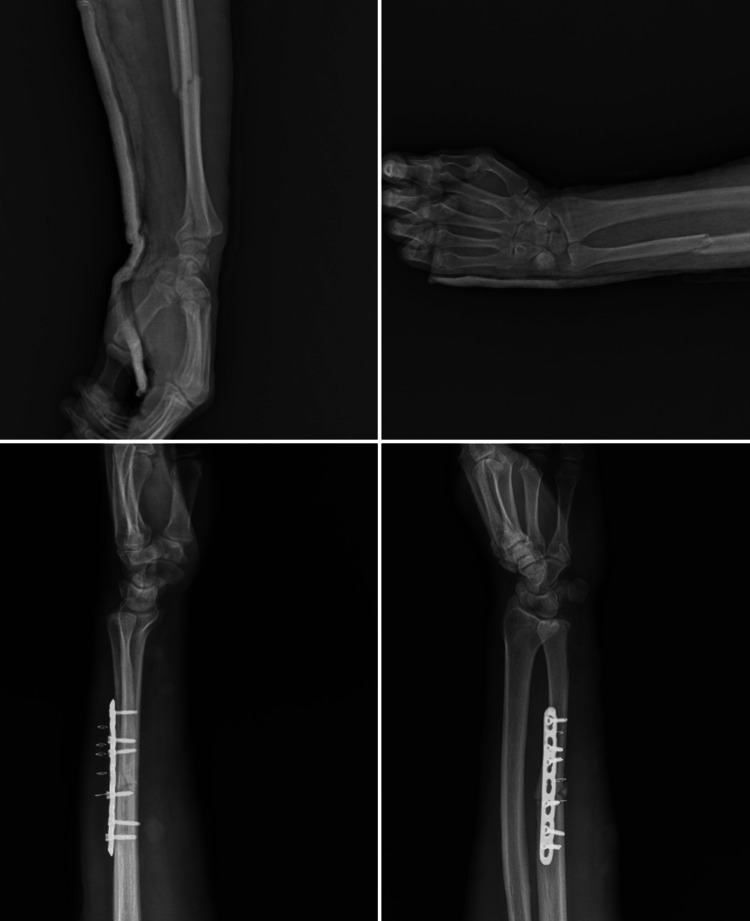
Preoperative and postoperative X-ray images of the patient.

**Figure 2:**
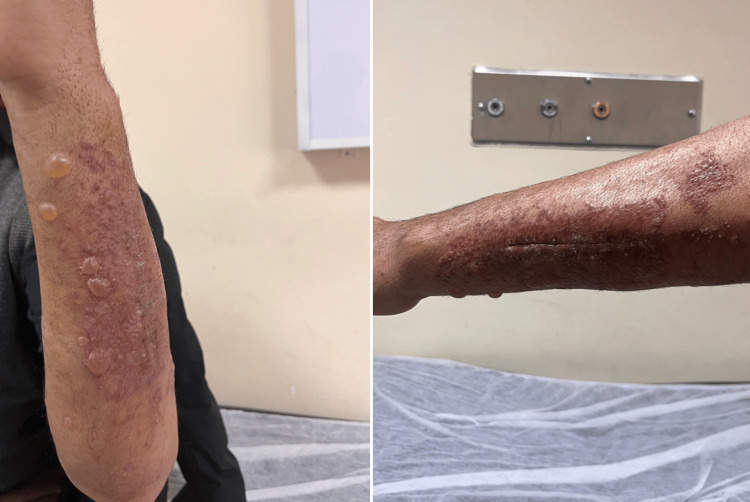
View of the patient's forearm after casting.

**Figure 3:**
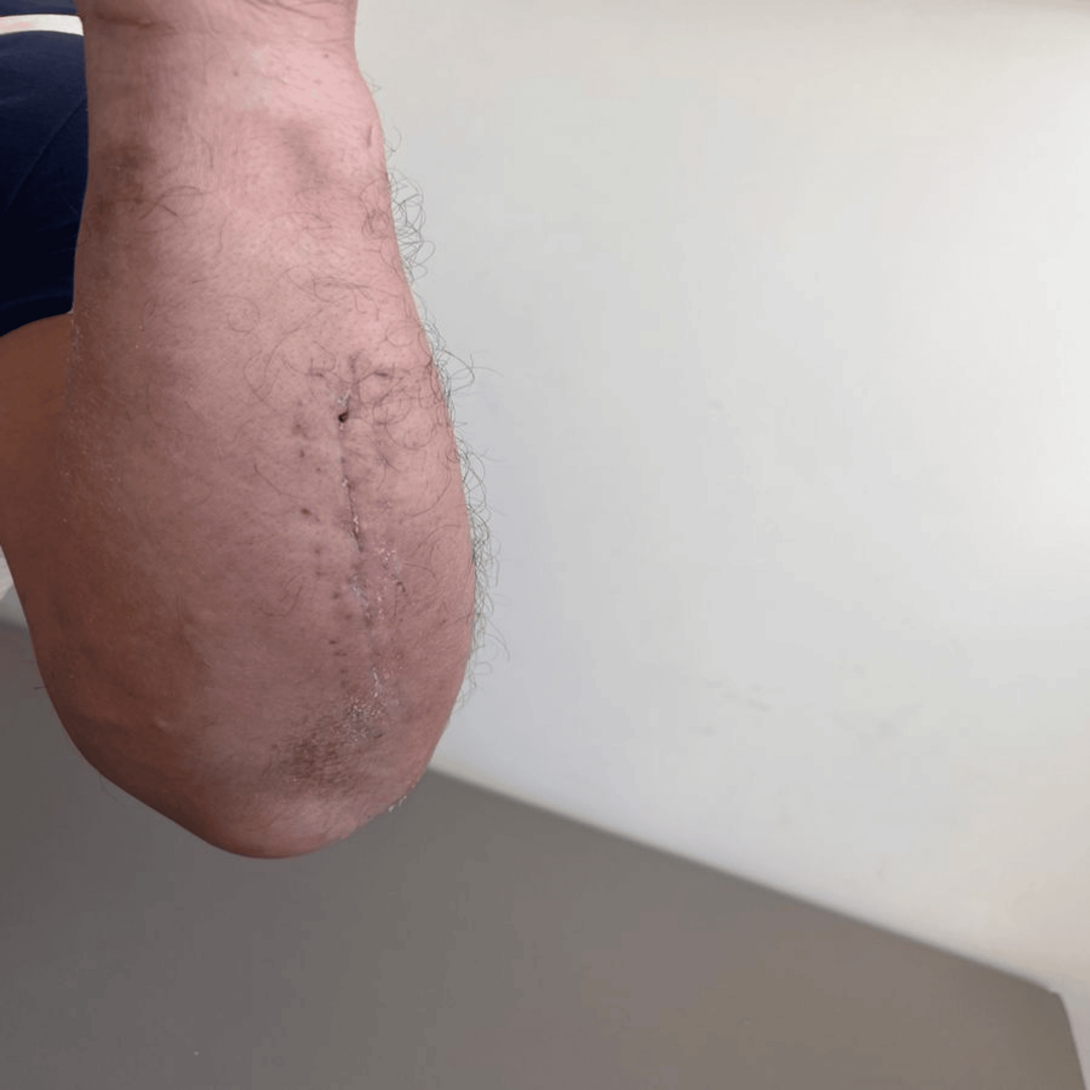
View of the forearm after casting removal, steroid, and antihistamine treatment.

A 52-year-old male patient presented to our outpatient clinic complaining of pain, swelling, and bruising in his left forearm following a simple fall. An X-ray revealed a fracture of the left ulna shaft. Under general anesthesia, the patient underwent osteosynthesis with plate fixation to repair the fracture, and a long arm cast was applied postoperatively (Figure 1). The patient's general condition was good, the incision site was clean, and follow-up was satisfactory. He was discharged the day after surgery and instructed to return for a checkup on the 15th day. However, on the third day after surgery, the patient presented to our outpatient clinic with redness, itching, and widespread bullous lesions at the incision site (Figure 2). The patient's medical history revealed no prior atopic dermatitis, contact dermatitis, dyshidrotic dermatitis, or hypertrichosis. He was referred to the dermatology department and received a preliminary diagnosis of allergic contact dermatitis. A 14-day course of steroids (methylprednisolone 40 mg/day) and antihistamines was initiated. The cast was removed and replaced with a simple arm sling. Cold application and upper extremity elevation were continued. Significant improvement was observed by the end of two weeks (Figure 3). Subsequently, a patch test was performed at a higher-level center to confirm the diagnosis and identify the allergen responsible. The test revealed an allergic reaction to synthetic cotton material. Immobilization was maintained using a simple arm sling during follow-up visits. At the end of the first month, active range-of-motion exercises for the forearm, elbow, and wrist were initiated. Functional recovery was fully achieved by the end of the third month, and bone union was completed at the end of the sixth month.

## Discussion

Casting is a treatment method frequently used in orthopedic clinics. However, many complications have been reported after casting, such as neurovascular damage due to circulatory disorders, lacerations, compartment syndrome, thermal burns, and pressure sores [1-4]. Nevertheless, there are only a limited number of studies on ACD formation. This case presentation is one of the rare reports describing ACD development after casting as a complication.

ACD localized to the plastered area may be caused by contact with components of the casting materials [4-8], and this may vary depending on the brand. It has been reported that ACD can be caused by chemicals that may be present in casts, such as benzalkonium chloride [5, 6], isocyanate [7], and formaldehyde [8]. Değer G et al. [9] reported the development of ACD during cast application after a distal radius fracture. The cast material used in the present case contained calcium sulfate, which is found in the synthetic cotton used. Fiberglass casts may be a suitable alternative in such cases, as they offer advantages such as higher porosity compared to other casts and reduced skin sensitivity to irritation beneath the cast [10].

## Conclusions

Consequently, the possibility of ACD development should be kept in mind when using materials for casting, and it should be noted that even the casting cotton used can cause this condition. Clinicians should also be aware that early removal of the cast may prevent further complications such as infection or delayed wound healing.
